# Preventing physical abuse of nursing home residents‐ as seen from the nursing staff's perspective

**DOI:** 10.1002/nop2.98

**Published:** 2017-09-14

**Authors:** Kjersti Lisbeth Braaten, Wenche Malmedal

**Affiliations:** ^1^ Department of Health and Welfare Trondheim Municipality Trondheim Norway; ^2^ Department of Public Health and Nursing Norwegian University of Technology and Science Trondheim Norway

**Keywords:** focus group interviews, nursing home, physical abuse, prevention, resident

## Abstract

**Aim:**

This study aims to capture first‐hand information from nursing home staff's own understanding regarding what they think and have experienced about prevention of physical abuse of nursing home residents and what measures they consider useful to implement in their daily work.

**Design:**

The design is qualitative.

**Methods:**

A convenient sample of staff in three nursing homes was used and data were collected during three focus group interviews. The total number of informants was 14. Thematic content analysis was used. The data collection period was from December 2015–February 2016.

**Results:**

According to the staff, several factors contribute to the prevention of physical abuse of residents in nursing homes. There is a requirement for increased competence among staff about the concept of abuse and known risk factors. Good communication skills and trusting relationships are important factors, as well as a culture that fosters openness where ethical dilemmas can be discussed.

## 1 INTRODUCTION

### 1.1 Background

Elder abuse is recognized internationally as a severe and widespread problem (World Health Organization, 2008), to which it is urgent to draw the attention of politicians, the health and care system and the public (Pillemer, Burnes, Riffin & Lachs, 2016). As the population steadily grows older, the coming decades will bring an increased number of older people who will require long‐term care (WHO, 2008). On this basis, it is likely that the incidence of abuse of nursing home residents will also increase (Pillemer et al., 2016). There is evidence that abuse and neglect are part of daily life in nursing homes in many countries (Bužgová & Ivanová, 2009; Goergen, 2001; Pillemer & Moore, 1989; Saveman, Astrom, Bucht & Norberg, 1999; Wierucka & Goodridge, 1996) and has been found to occur in Norwegian nursing homes (Hofseth & Norvoll, 2003; Kirkevold & Engedal, 2008; Malmedal, 1998; Malmedal, Hammervold & Saveman, 2014; Malmedal, Ingebrigtsen & Saveman, 2009).

The World Health Organization (2002) gives the following definition on elder abuse: “A single or repeated act, or lack of appropriate action, occurring in any relationship where there is an expectation of trust, which causes harm or distress to an older person” (p.3). Abuse can occur in various forms: physical, psychological, sexual, financial/material, or in the form of neglect. There are, however, no standardized or universal definitions of elder abuse (Norris, Fancey, Power & Ross, 2013). This is important to point out because how one defines the term and the method the researcher uses, will have an impact on the results obtained (De Donder et al., 2011).

Nursing home residents are particularly vulnerable to being subjected to abuse and neglect, due to their dependency on help from caregivers because of various illnesses (Malmedal, 2013). Different factors contribute to older people's vulnerability to abuse. These risk factors may include old age, cognitive decline/dementia, mental illness, physical impairment, being dependent on help and care and being socially isolated (Lachs & Pillemer, 2004; MacDonald et al., 2012; Pillemer et al., 2016).

According to Soares et al. (2010), old age causes reduced social network, loss of influence and higher dependency on other people due to several medical diagnoses. According to DeHart, Webb and Cornman (2009), medical diagnoses such as urinary tract infection, diabetes, or mental diagnoses, increase the likelihood of abuse due to misinterpretation of their behaviour as aggressive, e.g., diabetics can have fluctuating blood sugars that can have an impact on the way they behave and are interpreted by others.

Cognitive decline is a dominant symptom in more than 80% of nursing home residents. Dementia is the main cause, but depression, delirium, alcohol or various immune diseases can also lead to cognitive decline. It is important to note that these are treatable conditions, unlike dementia (Ranhoff, Smidt & Åstad, 2007).

It is crucial that staff are aware that dementia can lead to behavioural and psychiatric symptoms such as agitation, delusions, hallucinations and aggression, which may cause challenging behaviours. With staff shortages, turmoil and challenging behaviour may occur more frequently (Ranhoff et al., 2007). Studies show that staff knowledge about diseases and milieu therapy have an impact on the incidence of anxiety and acting out in people with dementia (Thorvik, Helleberg & Hauge, 2014).

Studies have shown that uncooperative and aggressive residents increase the risk of abuse (DeHart et al., 2009). A Norwegian study shows a clear correlation between resident aggression and physical abuse committed by staff (Malmedal et al., 2014). Other studies show that factors such as inadequate staffing, stressful workdays, time pressures, low wages and aggressive behaviour among residents may be related to abuse committed by staff in nursing homes (Pillemer et al., 2016).

Prevention of physical abuse of residents in nursing homes is an under‐researched topic (Malmedal, 2013; Pillemer et al., 2016). According to Pillemer et al. (2016) physical abuse is an intentional or unintentional action which causes physical pain or injury to another person. Physical abuse includes, but is not limited to, acts such as using force to hold a resident, restraint/holding back a resident, tying down a resident, holding a resident's nose to make him/her open their mouth, coerced/forced treatment (feeding, bathing, medication) (Malmedal, 2013). Unintentional abuse is when the perpetrator did not intend to hurt the person, but the act may have been the result of a lack of knowledge or experience (Kjølberg & Skaug, 2005).

Studies also show that detecting abuse can be problematic because it is hidden or ignored. The reason may be that the staff lack expertise and that residents are unable to speak out because of cognitive or physical limitations (Cohen, Halevy‐Levin, Gagin, Priltuzky & Friedman, 2010; Lachs & Pillemer, 2004; Thorvik et al., 2014).

To prevent abuse, one must first recognize that abuse actually occurs. According to Malmedal (2013) an important aspect of good practice is to be able to detect abuse and neglect and to understand how severe the consequences can be for residents.

Studies show that training that increases staff skills can give staff more expertise in recognizing and dealing with challenging ethical issues in everyday life. Furthermore, competence could help increase staff awareness and promote a better understanding of risk factors associated with physical abuse of nursing home residents (Daly & Coffey, 2010; DeHart et al., 2009; Malmedal, 2010). Very little is known about how staff in nursing homes perceive and prevent physical abuse of residents and this study will contribute to closing this knowledge gap.

### 1.2 Aim

This study aims to capture first‐hand information from nursing home staff's own understanding regarding what they think and experience with regard to prevention of physical abuse of nursing home residents and what measures they consider useful to implement in their daily work. The main question is: How can staff prevent physical abuse of nursing home residents? Answers will be sought through the following research questions:

1. In what way does the nursing home focus on prevention of physical abuse of residents?
2. What factors do staff consider to be important to prevent physical abuse of residents?
3. In what ways do staff reflect on whether their own practices promote abuse prevention?

## 2 THE STUDY

### 2.1 Design

The study design is qualitative and descriptive. When the aim is to illuminate attitudes and experiences, focus group interviews are considered a fruitful method (Krueger & Casey, 2009). The data is gained through interaction between participants involving a theme chosen by the researcher. Conversation between the informants about common experiences will add important insight for the researcher (Halkier, 2010). By choosing to collect data through conversation, the context around this conversation will be of great significance to the knowledge obtained. It is of great importance that the researcher has knowledge and understanding of the dynamics between the researcher and the informant and how this affects the analysis of the data material and the results of the study (Kvale & Brinkmann, 2009).

### 2.2 Participants

To recruit staff with experience from nursing homes to reflect on their own practice, the inclusion criteria for this study were male and female staff who had been permanently or temporarily employed for at least 1 year in one of the selected three nursing homes in one city in central Norway. Informants were recruited with help from nursing home managers who had consented for the nursing home to be included in this study. Fourteen participants volunteered for the study. All were female with an age range from 24‐53 years. Three were registered nurses at bachelor level, one social educator, also holding a bachelor qualification and the rest were licensed practical nurses (4) and healthcare workers (6) educated to high school level. All had more than 1 year of experience, with a range from 2‐20 years.

### 2.3 Data collection

To obtain a sufficient number of participants in this study, the interviews took place at their workplace during working hours. Contrary with conducting interviews in a neutral place, this could have been an advantage as the context for interviews related to the topic of study. The context may have made it easier to recall memories of past episodes and experiences in the same context (Josephsson & Alsaker, 2014). The focus group interviews were conducted in a meeting room at each of the participating nursing homes. A short movie from the e‐learning program “Elder abuse in nursing homes” (Vern for eldre), showing a nursing home resident, Oscar, as a victim of physical and psychological abuse by staff, served as an introduction to the topic. An interview guide that consisted of open‐ended questions related to the movie and to the research questions, allowed the participants to reflect and to talk freely; this guide also provided a checklist of key questions on the topic and an aid to the interviewer to keep the thread of the interview. At one of the interviews, both authors participated: KB as moderator and WM as co‐moderator. At the two remaining interviews, only KB participated. All three interviews were recorded. Five interviewees participated in two of the interviews and four in 1 interview, in total 14 participants. Each interview lasted approx. 45 min.

### 2.4 Data analysis

The recordings were transcribed verbatim by KB after each interview. This was because it is easier to remember the entire conversation shortly after. It was easy to distinguish the participants from each other, both through their distinct dialects and because they presented themselves at the beginning of the interview. The purpose of transcription is to capture what the conversation was all about and to reveal what informants say on a matter. When recordings are transcribed into text, differences may occasionally occur because dialectical expressions are translated to ensure informants' anonymity (Krueger & Casey, 2009; Malterud, 2011).

The text was analysed using systematic text condensation, as described in Malterud (2011). This is a step by step analytical method. Systematic text condensation is inspired by Giorgi's phenomenological analysis, which is a method that seeks to describe informants' experiences and their “life world”. It is all about finding out what characterizes a phenomena; hence its use in our research. It is important that the researcher is aware that the analysis will always consist of subjective interpretations. Systematics, thoroughness and a thorough and well‐documented analysis are needed for the credibility of the research (Kvale & Brinkmann, 2009; Malterud, 2011).

A thorough reading of the transcribed interviews identified four main themes and meaningful units. After going through each meaningful unit, three subgroups were identified under each meaningful unit. Eventually, four categories developed from the essence of each code groups were summarized. It is important to note that even if something is said repeatedly by several informants, it does not necessarily mean that these are the most important findings. Often, something that is expressed just once happens to be very interesting (Krueger & Casey, 2009; Malterud, 2011).

It is of great importance that the researcher is critical to one`s own role in the research process, so that the result could be perceived as reliable (Malterud, 2012). It is also important that the researcher is able to reflect critically around which transferability the findings have beyond that context where the research have been conducted (Malterud, 2012).

### 2.5 Ethical consideration

The study is approved by The Privacy Issue Unit of the Norwegian Social Science Data Services (Reference no. 43748) Prior to the study, participants were given thorough information via a letter which described the purpose of the study and a request to participate in the research project. Participants were assured full anonymity and information has been de‐identified and is not recognizable in the results section. It was emphasized that participating in the study was voluntary and the participants were told that they had the opportunity to withdraw from the study at any time without explanation and with no consequences. Each participant signed a consent form prior to the interview.

### 2.6 Methodological considerations

There are advantages and disadvantages of using focus group interviews as a data collection method. Focus groups are well suited to describe opinions and experiences among health workers (Malterud, 2012). Group discussions have many benefits: the participants can help each other to express their views. If participants are colleagues, as in this study, they already know each other and probably feel safe together. On the other hand, they may be reluctant to speak in a group because they are afraid of saying something that might be perceived as wrong. The use of focus groups was considered an appropriate method for this study and the response from the participants confirmed this. Three focus group interviews with a total of 14 participants were conducted in this study. According to Malterud (2011), a researcher will have reached saturation point when they find that additional data does not provide new information (Malterud, 2011). In this study, the initial plan was to conduct two group interviews, but one extra was added to see if any new information emerged. The informants said much the same in all the interviews, so the saturation point was reached after three focus group interviews. The nursing homes in the study differed in size and location and we thus believe that the results are valid for other nursing homes as well. The study has however some limitations that has to be mentioned. The homogeneity of the group may have limited a more nuanced insight into the topic, since all participants were female staff, with an age range from mid‐30s to 50s. Older staff and male staff might have other experiences that would be worth studying.

## 3 FINDINGS AND DISCUSSION

### 3.1 Main findings

The three focus group interviews revealed findings that in some areas were similar, but differences also emerged. The definitions used in this study does not limit physical abuse to be acts committed by staff only and the participants were free to discuss other groups of perpetrators; co‐residents, relatives, other visitors. However, they mostly discussed episodes when staff were involved in incidents that is defined as abuse. The analysis resulted in four main findings that reflect the informants' views about what prevents physical abuse against nursing home residents. These key findings were communication, building trust, skills and competence and the work environment. Each main finding was then divided into three subgroups. The main finding, “communication”, was divided into personal chemistry, teamwork and ethical reflection. “Building trust” was divided into relationships, user involvement and relatives. “Skills and competence” was divided into the subgroups knowledge, management and documentation, while “work environment” was divided into the subgroups permanent versus temporary staff, culture and pace.

### 3.2 Detecting physical abuse in nursing homes

At the beginning of each focus group interview, the participants watched a movie of a patient case, as presented earlier. This proved to be a good basis for discussion in the group. Even though all the participants judged the situation in the film to be of an abusive nature, they claimed that in practice it was often difficult to say whether an act was abusive or not. “It is a dilemma when there are so many gray zones,” said one of the interviewees. Another in the same focus group continued, “Abuse can happen daily and it is therefore important to focus on it almost every day, to prevent it from happening.” Research shows that health workers have little knowledge about elder abuse (Helse‐og omsorgsdepartementet, 2012) and it is important that staff receive training concerning elder abuse and knowledge about the risk factors (Malmedal, 2013; Pillemer et al., 2016). In response to the question, “So how do you draw a line between what is and is not physical abuse?” one of the informants said, “We must listen to the resident! If he says stop, we must stop! In addition, we need to find out why he says stop!” The difficulty knowing when you are crossing someone's boundaries was mentioned several times in all three groups. The informants stressed that it is important to be aware that there are gray zones, because the helpers will then be more cautious. One of the participants summed up the discussion: “Physical abuse should not happen, because you've got in mind what it is. I feel it takes very little before the line is crossed!” An inherent balance of power exists between vulnerable nursing home residents and staff. The residents rely on help and this gives staff power over residents, a power that should be used in a responsible way (DeHart et al., 2009). It is important to be conscious of this to prevent abuse. Another interesting aspect in this context is that dependency on help is a strong risk factor for elder abuse (Pillemer et al., 2016). In Norwegian nursing homes, more than 80% of the residents suffer from dementia (Selbæk, Kirkevold & Engedal, 2007), which is a known risk factor for abuse (Pillemer et al., 2016).

### 3.3 Communication

Health professionals need good communication skills to prevent conflicts that may lead to abuse (DeHart et al., 2009). The informants pointed out the necessity of using communication actively in the relationships. One informant expressed, “It is important to always inform about what you plan to do in advance, the resident will feel safer then.” Cognitive impairment can lead to huge challenges in communication. This applies both to understanding what is said and being able to express one's wishes (Engedal, 2008). In this context it is likely that communication difficulties may lead to the risk of abuse. Health professionals must be aware of both verbal and non‐verbal communication with residents, to reduce conflicts and prevent abuse (DeHart et al., 2009).

### 3.4 Personal chemistry

One aspect of communication that informants were concerned about was the personal chemistry between the staff and the resident: “Some people just have better chemistry than others” or “You do not have chemistry with everyone,” was echoed by several informants. The participants believed that good chemistry between staff and residents can help to prevent physical abuse of residents, because people who feel attached to each other understand each other better. In this context, it is likely that the chemistry between people is associated with both communication and relationships. DeHart et al. (2009) emphasizes that the ability to communicate effectively with nursing home residents can be learned. Good chemistry between people is associated with the ability to communicate well and appropriately and to show good judgement in relationships with residents. To show good judgement as a health worker, it is essential that they use their knowledge of communication, ethics and experience and reflect on these (Nortvedt, 2012).

### 3.5 Teamwork

Another aspect of communication is cooperation between colleagues. Several informants expressed that it should not be considered a failure to ask a colleague for assistance. One informant stated, “If I fail to help a resident, maybe someone else can try?” Developing a work environment with good cooperation between workmates, can help to prevent abuse of nursing home residents (DeHart et al., 2009). Colleagues can help each other when conflicts arise, or if a colleague is under great pressure at work and in this way prevent abuse (DeHart et al., 2009).

### 3.6 Ethical reflection

A third aspect of communication is ethical reflection. Several informants stressed that good communication between colleagues also meant that one could talk about dilemmas and disagreements. One informant said, “I feel that we bump into ethical dilemmas every day and it is important to have time to discuss with colleagues to come up with good solutions.” One of the informants said, “With regard to abuse it is important to have a culture where colleagues can talk together and engage in ethical reflection”. One of the informants said, “By reflecting ethically together we force ourselves to think things through in a different way in a hectic work day.” The informants also mentioned the importance of staff being aware of their body language in front of the resident. One of the informants said, “It is important to reflect about the way you meet a resident. What you say and how you behave!” It can be assumed that it is not always easy for care workers to be conscious of the impact of their own communication. In this context, one would think that the ability to ethically reflect together with colleagues and be constructively critical of their own practices could be a way to prevent physical abuse. It is an important task for the management of nursing homes, to encourage staff to engage in ethical reflection (Daly & Coffey, 2010; Malmedal, 2013).

### 3.7 Building trust

Several participants expressed the importance of building trust to avoid abuse. One of the informants expressed how there is more focus now on trust‐building work than before, even in relation to residents where you can use restraint/force and that there has been an increased awareness that trust‐building measures should always be tried first.

### 3.8 Relationships

One aspect of trust is good relationships. Many of the participants expressed that it is necessary to be aware of how important it is that the resident feels trust in the relationship. Trust can help increase the resident's tolerance to closeness (Hummelvoll, 2012). According to the informants, it is essential that the resident should not have to deal with a wide range of people, to easier build good relationships. Several of the informants believe that you build a good relationship by showing interest in the resident. The resident will then feel seen, heard and respected. One informant said: “Health professionals should have a “care gene” that lets the elderly retain control over their own lives. When we use this and are allowed to help, a lovely interplay arises.” Interpersonal skills are about health care workers showing their capacity for good and appropriate communication with the nursing home residents they care for. The health care worker must be patient as it takes time to build a good relationship. Research shows that good relationships can help to reduce the risk of aggression between staff and residents, because staff are more likely to show greater respect for residents' preferences in a good relationship (Bužgová & Ivanová, 2009). Another study shows that it is much more difficult to abuse someone with whom you have a good relationship and you care about (DeHart et al., 2009).

### 3.9 User involvement

Another aspect of building trust is user involvement. Several informants expressed that health professionals need to have a lot of patience, insight and empathy, as well as take seriously the resident's desire for independence. Several informants had experienced how one could prevent physical abuse by arrangement with the residents about when it suits them to receive help. One informant expressed: “It must not happen then and there! If you cannot make it then, we can come back later, or ask a colleague for help.” A study by DeHart et al. (2009) shows that it is important to have a deliberate strategy to prevent abuse. One could, for example, come back later, offer choices, or ask for help from a colleague if the resident is behaving aggressively. Moreover, it was highlighted in the interviews that staff should ask residents about why they did not want help. One of the informants said: “We must find out why the resident does not want help. Is there anything he is withholding? Is he in pain? Is it anxiety?” The informants discussed the importance of primary contacts. They concluded that by having primary contacts you can build good relationships more easily, which can create security and predictability for the resident. Some informants talked about how being creative in a situation could prevent physical abuse, for example to implement music to decrease agitation and to calm down the situation. Cognitive impairment or dementia can cause major problems with communications. It may be useful to communicate in other ways, for example through music (Myskja, 2009). Research shows that use of music is proven to have an effect on the incidence of agitation and unrest among nursing home residents (Korb, 1998).

### 3.10 Relatives

A third aspect of building trust is related to engaging relatives as a resource. According to the informants, by having a good relationship with relatives, staff can learn more about the resident and hear about how the resident has lived his/her life. “When the staff maps the life story of the resident, then we'll know more about them and it is perhaps easier to meet them in a good way”, said one informant. According to Malmedal (2013), it is essential that staff build good relationships with nursing home residents' relatives. Isolation and lack of contact with the outside world is a risk factor for physical abuse in nursing home residents (Lachs & Pillemer, 2004; MacDonald et al., 2012; Pillemer et al., 2016). Nursing homes that promote an open culture, where families feel welcome, can help to prevent abuse (Malmedal, 2013). However, research shows that older persons may be vulnerable to abuse from relatives (Sandmoe & Skraastad, 2013). It is therefore important staff know that although living in nursing homes, the resident could still be abused by relatives during visits. One of the informants said: “Healthcare professionals should be aware that relatives can cross the residents' boundaries. It is important that staff are able to intervene and bring about a dialogue in relation to what is appropriate behaviour.”

### 3.11 Skills and competence

All focus groups discussed the importance of highly skilled staff to prevent physical abuse. One informant said: “By increasing our expertise, we understand better how to help residents.” Research shows that there is a correlation between low skills and the risk of abuse of nursing home residents (Sandvide, Åström, Nordberg & Saveman, 2004).

### 3.12 Knowledge

One aspect related to skills and competence is knowledge. A study by Daly and Coffey (2010) shows that there is a high degree of uncertainty among staff about what abuse actually is and there is a need for knowledge among health workers on this topic. The participants in the focus group interviews sometimes found it hard to judge whether to label an act as abuse or not. Another factor that appeared in the interviews is the importance of training new employees. A study by DeHart et al. (2009) shows that knowledge is needed to prevent abuse and that training contributes to increasing staff awareness of abuse, as well as their understanding of risk factors. The focus groups discussed how staff could increase their knowledge and one informant said: “By taking a course, we get a lot of tools to avoid using force and avoid disruptive behaviour.” Informants indicated that a larger proportion of staff at the nursing home should be of a higher educational level, because education and training can facilitate more in‐depth understanding and expertise among the staff. Research shows that training and education of staff may increase the ability to recognize abuse situations and handle conflicts (Malmedal, 2010). In one focus group the Marte Meo method was mentioned as an aid to prevent physical abuse. Marte Meo is a relationship‐oriented method – for use in practice – that creates awareness both in the individual health worker regarding their own behaviour and in staff as a team about how, together, they can implement successful measures. Furthermore, it also focuses on perceiving signals from residents at an earlier stage (Hatløy & Alnes, 2007). By taking cues from residents at an early stage, it is reasonable to believe that conflicts and abuse can be more easily avoided. The informants in the focus group were trained in the method and had adopted it, with good results. One of the informants said: “The method is worth gold. We have used it many times in the nursing home.” Studies show that by adopting a relationship‐oriented method such as Marte Meo, staff are better at perceiving signals from the resident at an earlier stage (Hatløy & Alnes, 2007).

### 3.13 Management

Another aspect related to skills and competence is leadership. Management is identified as an important factor to improve quality and to enhance patient safety in nursing homes (Blouin & Buturusis, 2012; Siegel, Mueller, Anderson & Dellefield, 2010). Informants indicated that they saw the leaders as important role models in relation to attitudes and values. One said: “It is important to have an open working environment so employees can talk about mistakes that are made or if the patients' safety is threatened.” In a study undertaken by DeHart et al. (2009), lack of management supervision was listed as one factor that could increase the risk of abuse of nursing home residents. One focus groups stated that the management of the nursing home should take responsibility for ensuring that employees had the opportunity to take courses and gain higher qualification. One of the informants said: “More funds should have been set aside and invested in training and skills development; how else can we manage with high levels of sick leave and a tight economy?”

### 3.14 Documentation

A third aspect relating to skills and competence is documentation. Staff need to cooperate to help the residents in the best way and several informants said that documentation must be part of this cooperation to prevent physical abuse. By documenting how various measures and methods work, the information transfers to colleagues for the benefit of the resident. One informant expressed: “It is crucial to be conscious of how we document and that we follow‐up documentation.” In this context, it is important for staff to be aware of documented measures regarding aggressive behaviour that have effect and use this information to prevent abuse.

### 3.15 Work environment

It is important that a workplace is organized in such a way that respecting nursing home residents is given priority and that ethical principles are adopted (Bužgová & Ivanová, 2009). Studies show that many health workers are not aware that their behaviour toward nursing home residents would be considered physical abuse (Sandvide et al., 2004).

### 3.16 Permanent versus temporary staff

One aspect related to the work environment is use of permanent staff as opposed to temporary workers. Several informants indicated the importance of who is on shift, since staff that are familiar with the residents, can keep the unit calmer. One of the informants said: “We find the residents act out more and there is more use of force and restraint when those at work do not know the residents and thus do not know how to handle them.”

### 3.17 Culture

Another aspect related to the work environment is whether there is a good or bad culture. It may, according to the informants, be difficult to provide appropriate training for new employees if a culture of safety is missing from the workplace. If long‐term, permanent employees talk to residents in a disrespectful way, new employees tend to adopt this behaviour, according to one informant. Studies show that workplace culture is crucial to the degree to which abuse occurs (Pillemer, 1988). One of the participants expressed: “In order to prevent abuse, it is important to dare to speak up about bad culture.” Another informant stated that this depends on the personality of each individual and that it may be easier for staff with a higher level of education and more experience to speak out. Research shows that one must feel that speaking out will make a difference (Hetle, 2005). If one is afraid of possible consequences, such as losing a job or being seen as disloyal toward colleagues, one may not dare to tell (Calcraft, 2005). If the focus is on who is to blame, at worst this could lead to a situation where no one is prepared to report instances of abuse (Malmedal, 2013). It is important to work toward an open and positive work environment, where there is room to discuss and reflect on individual practices and not only when there are challenging situations (Calcraft, 2005).

### 3.18 Pace

A third aspect related to work environment is pace. The focus groups discussed the necessity of planning the day from the perspective of the resident. “If you know there is a resident you need to spend more time with, to help him/her, you must set aside enough time for this person,” said one of the informants. The participants agreed that if staff slowed down, the result was actually better. One of the informants expressed: “If you use five minutes extra on your morning routine, the resident will be calmer for the rest of the day.” However, it may be difficult to slow down because of time pressures. Several informants claimed that by giving the resident a quiet start to the day, one could prevent physical abuse. “Some residents may want to sleep longer and some may want breakfast in bed,” one informant said. “So it is all about facilitating individual needs.” Use of person‐centred care can meet individual needs (Malmedal, 2013). By adopting person centeredness in the care of nursing home residents with dementia or cognitive impairment, staff can more easily communicate with these residents and thus reduce aggressive behaviour (Enmarker, Olsen & Hellzen, 2011). In this context, one can assume that adopting methods that promote communication between staff and residents could prevent physical abuse.

## 4 CONCLUSION

Research addressing prevention of physical abuse of nursing home residents is very sparse (DeHart et al., 2009; Pillemer et al., 2016). Research shows, however, that measures implemented should aim to reduce risk factors for abuse (Malmedal, 2013; Pillemer et al., 2016). Findings from the focus group interviews showed that staff were able to point out important areas that could be focused on, to prevent physical abuse in nursing homes. It is important to increase nursing staff competence in identifying abuse and known risk factors. It is further necessary to make efforts to prevent conflicts. Findings from this study indicate that effective communication and teamwork are important factors. Knowing the resident may decrease the risk of abuse, since it is less likely that health workers will carry out assaults on residents they have close and good relations with (DeHart et al., 2009). Staff should be aware of the significance of good and appropriate communication and in addition, the need to reflect on their own behaviour and communication skills. It is also important to work toward a culture that recognizes the necessity of looking critically at their own practices, as well as engendering an openness where you can discuss ethical dilemmas. It is thus necessary to set aside time for ethical reflection in a hectic schedule, which can help to create an awareness among staff about their own behaviours and mindsets.

It is also important for health workers to work in such a way that maintains the residents' autonomy. By using a person‐centred approach in care, staff can move the focus from the disease itself to see the person with the disease (Kitwood, 1997). In this way, one could argue that the resident may feel seen and recognized by the staff, which in turn could help to build a stronger relationship. By adopting person‐centred care, staff attitudes can change. It is all about interpreting the behaviour of the residents, regardless of how this behaviour appears (Malmedal, 2013). Furthermore, it is important that staff see the relatives as a resource. Relatives can help staff to become better acquainted with the resident and to combat the isolation that may be a risk of abuse.

An awareness of the topic among health professionals and in society in general is essential, to prevent abuse. It is imperative that policies and procedures for how health workers should handle abuse in nursing homes are developed.

## AUTHOR CONTRIBUTIONS

Study design: KB, WM; data collection: KB, WM; data analysis: KB, WM; manuscript preparation: KB, WM. Both authors have agreed on the final version. There are no conflicts of interest and no funding is received for this study.
